# Isolated auditory hallucinations as the initial neuropsychiatric manifestation of dementia with Lewy bodies: diagnostic pathway and individualized therapy (a case report)

**DOI:** 10.3389/fpsyt.2026.1935514

**Published:** 2026-09-11

**Authors:** Lijun Hou, Xingmei Ma, Chunfang Wang, Lan Ji, Peiying Peng

**Affiliations:** 1 Department of Neurology, Qujing Second People’s Hospital, Qujing, Yunnan, China; 2 Qujing Medical College, Qujing, Yunnan, China

**Keywords:** antipsychotic sensitivity, dementia with Lewy bodies, integrated diagnostic strategy, isolated auditory hallucinations, rapid eye movement (REM) sleep behavior disorder

## Abstract

Dementia with Lewy bodies (DLB) is characterized by visual hallucinations. Isolated auditory hallucinations as the presenting psychiatric symptom are uncommon, and misdiagnosis as a primary psychiatric disorder with consequent inappropriate antipsychotic exposure is a substantial risk. A case of probable DLB presenting with isolated auditory hallucinations as the initial manifestation, accompanied by RBD, parkinsonism, and characteristic neuroimaging findings, is described. Initial misdiagnosis as schizophrenia led to severe adverse reactions to antipsychotic agents. After multimodal evaluation, treatment with a cholinesterase inhibitor, combined with a monoamine oxidase-B inhibitor and a melatonin receptor agonist, improved motor and sleep symptoms, and the patient’s condition gradually stabilized. Although isolated auditory hallucinations are not a typical presenting feature of DLB, they should be regarded as a warning signal for α-synucleinopathy when occurring against a background of neurodegeneration accompanied by RBD, autonomic dysfunction, or Parkinsonism. When clinical features satisfy the criteria for probable DLB, core clinical features are sufficient to establish the diagnosis; cholinesterase inhibitors should be the first-line treatment for neuropsychiatric symptoms in DLB.

## 1 Introduction

Dementia with Lewy bodies (DLB) is characterized by fluctuating cognition, visual hallucinations, parkinsonism, and rapid eye movement (REM) sleep behavior disorder (RBD) (1, 2). Neuropsychiatric symptoms develop in approximately 75% of patients during the disease course; however, isolated auditory hallucinations as the inaugural manifestation are exceedingly rare. A systematic review demonstrated that pure auditory hallucinations account for merely 0.6% of DLB cases (3), a low prevalence that renders affected patients vulnerable to misdiagnosis as primary psychiatric disorders and consequent inappropriate antipsychotic exposure with characteristic drug-related adverse reactions in DLB (4).

The 2017 DLB Consortium criteria stipulate that dementia plus ≥2 core clinical features establishes a diagnosis of probable DLB; indicative biomarkers serve only to increase diagnostic certainty rather than being mandatory (2). Herein, we report a patient with DLB who presented with isolated auditory hallucinations and experienced a 6-year diagnostic delay. We explore the pathophysiological mechanisms underlying atypical inaugural neuropsychiatric symptoms, the decisive role of core clinical features, the differential diagnostic value of multimodal investigations, and safe management strategies for neuropsychiatric symptoms in DLB.

## 2 Case presentation

A 59-year-old male engineer with 16 years of formal education denied any family history of neurological or psychiatric disorders, as well as smoking and alcohol consumption. He lived with his wife, who served as the primary caregiver. No significant psychosocial stressors preceded symptom onset. Hyposmia had developed several years prior to the index presentation but was disregarded. He was referred to our clinic in June 2025 with complaints of “recurrent auditory hallucinations for 6 years, bradykinesia for 4 years, and visual hallucinations for 2 years.”

**Figure 1:**
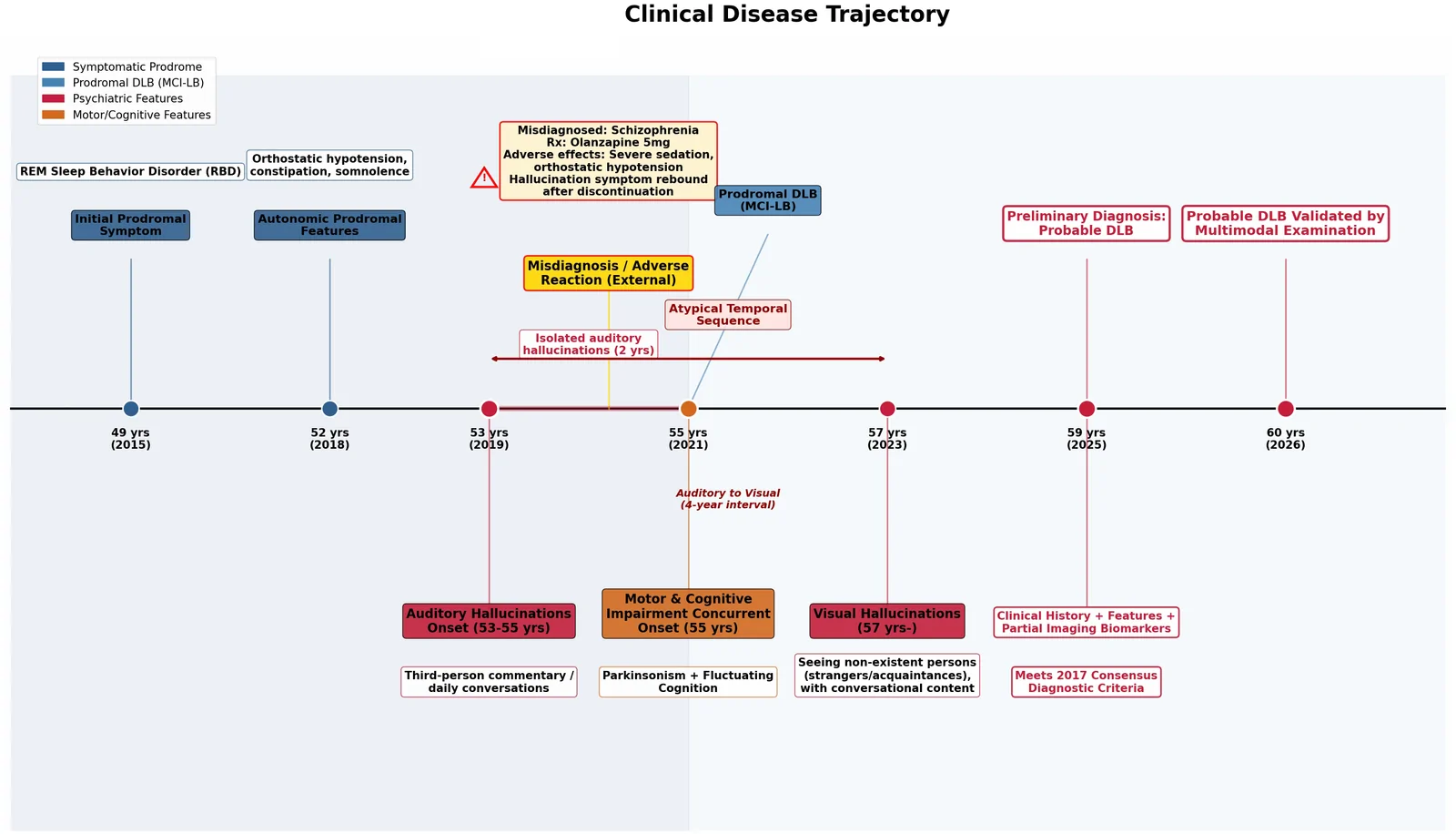
Clinical trajectory from prodromal symptoms to probable dementia with Lewy bodies (DLB) diagnosis (ages 49–60 years). Prodromal manifestations included REM sleep behavior disorder at age 49 years and autonomic dysfunction at age 52 years. Auditory hallucinations emerged at age 53 years and persisted for 2 years, followed by parkinsonism with fluctuating cognition at age 55 years. Visual hallucinations developed at age 57 years, constituting an atypical temporal sequence with a 4-year interval between auditory and visual onset. The patient was misdiagnosed with schizophrenia and treated with olanzapine, which caused sensitivity-related adverse effects. Preliminary probable DLB diagnosis at age 59; validated by multimodal examination at age 60.

Symptom chronology is reconstructed as follows (**Figure 1**). At age 49, sleep-related vocalizations and violent limb movements emerged, resulting in his wife’s injury as the bed-partner. Around age 52, he developed excessive daytime sleepiness (afternoon naps lasting up to 2 hours), somnolence during seated reading, and nocturnal sleep-onset insomnia. Concurrently, recurrent orthostatic dizziness-confirmed by the attending physician as orthostatic hypotension-and chronic constipation emerged. At age 53, isolated auditory hallucinations first appeared: clear conversational voices discussing work and daily life, without commenting or commanding features, initially occurring 1–2 times monthly and progressing to 2–3 times weekly; insight was absent. At age 55, bradykinesia, resting tremor, and rigidity appeared, accompanied by progressive cognitive decline. His wife reported fluctuating cognition characterized by sudden staring episodes and inappropriate responses lasting minutes to hours, increasing from 3–4 episodes weekly to 1–2 daily. He took early retirement due to symptom progression. At age 57, visual hallucinations developed (1–3 episodes weekly), consisting of nonexistent individuals (strangers or acquaintances) with conversational content.

Prior Treatment History: At the onset of auditory hallucinations in 2019, he was misdiagnosed with schizophrenia at another institution and prescribed olanzapine 5 mg nightly. Although hallucinations transiently remitted, severe sedation (retrospective Epworth Sleepiness Scale score increased from 10 to 18) and symptomatic orthostatic hypotension occurred within 2 days, necessitating drug discontinuation. No further antipsychotic agents were administered thereafter. (Note: Schizophrenia rarely manifests as a first episode after age 53; this age characteristic should serve as a critical warning signal for organic etiologies (5).

Neurological Examination: Masked facies, cogwheel rigidity, resting tremor, and orthostatic hypotension were present. Bedside olfactory testing (mint, coffee, clove) indicated hyposmia. Cerebellar signs were absent. The baseline Unified Parkinson’s Disease Rating Scale Part III (UPDRS-III) score was 27. An acute levodopa challenge test was performed per standard protocol using levodopa/carbidopa 100/25 mg orally (6), with UPDRS-III assessments at baseline and 1, 2, and 3 hours post-administration. The maximal improvement was 11.1% (UPDRS-III score 24 at 2 hours), indicating a poor levodopa response.

Neuropsychological Assessment: The Mini-Mental State Examination (MMSE) score was 23/30. Adjusted for the patient’s educational background, this score was consistent with mild cognitive impairment or early-stage dementia. The Montreal Cognitive Assessment (MoCA) score was 19/30, below the normal cutoff of 26. The MoCA is more sensitive than the MMSE in assessing executive function and visuospatial abilities; the results aligned with the characteristic cognitive profile of DLB, in which early visuospatial and executive deficits predominate over memory impairment. The Mayo Fluctuation Scale score was 3, suggestive of significant cognitive fluctuations—a core clinical feature of DLB. The Epworth Sleepiness Scale score was 14, indicating moderate daytime somnolence (>10 is considered abnormal), which concurred with the clinical presentation of RBD. The Barthel Index was 100/100, whereas the Instrumental Activities of Daily Living (IADL) scale score was 4/8. Taken together, these findings suggest that, although the patient retained intact basic activities of daily living, instrumental functioning related to complex cognition was already impaired. See **Table 1**.

**Table 1:** Diagnostic concordance matrix: correlation between clinical features and objective biomarkers.

Diagnostic level	Clinical manifestations	Status
Basic Feature	Progressive cognitive decline affecting instrumental daily function; early visuospatial/executive dysfunction predominating over memory impairment. MMSE 23/30, MoCA 19/30, IADL 4/8.	Fulfilled
Core Clinical Features	1. Fluctuating cognition: 1–2 daily ‘blank mind’ episodes, Mayo Fluctuation Composite Scale 3 points. 2. Recurrent visual hallucinations: seeing nonexistent people from age 57. 3. REM sleep behavior disorder: dream-enactment behavior from age 49 (prodromal). 4. Parkinsonism: bradykinesia, tremor, rigidity from age 55; poor levodopa response.	Fulfilled 4/4
Supportive Clinical Features	Antipsychotic sensitivity: olanzapine-induced hypersomnia and orthostatic hypotension. Autonomic dysfunction (orthostatic hypotension, constipation). Excessive daytime sleepiness. Hyposmia.	Fulfilled

RBD, rapid eye movement sleep behavior disorder. Diagnostic levels are based on the 2017 DLB Consortium consensus criteria (McKeith et al, 2017). Epworth 10 indicates the patient’s retrospective estimated baseline score before olanzapine use.

## 3 Diagnostic assessment, therapeutic intervention, and follow-up outcomes

**Figure 2:**
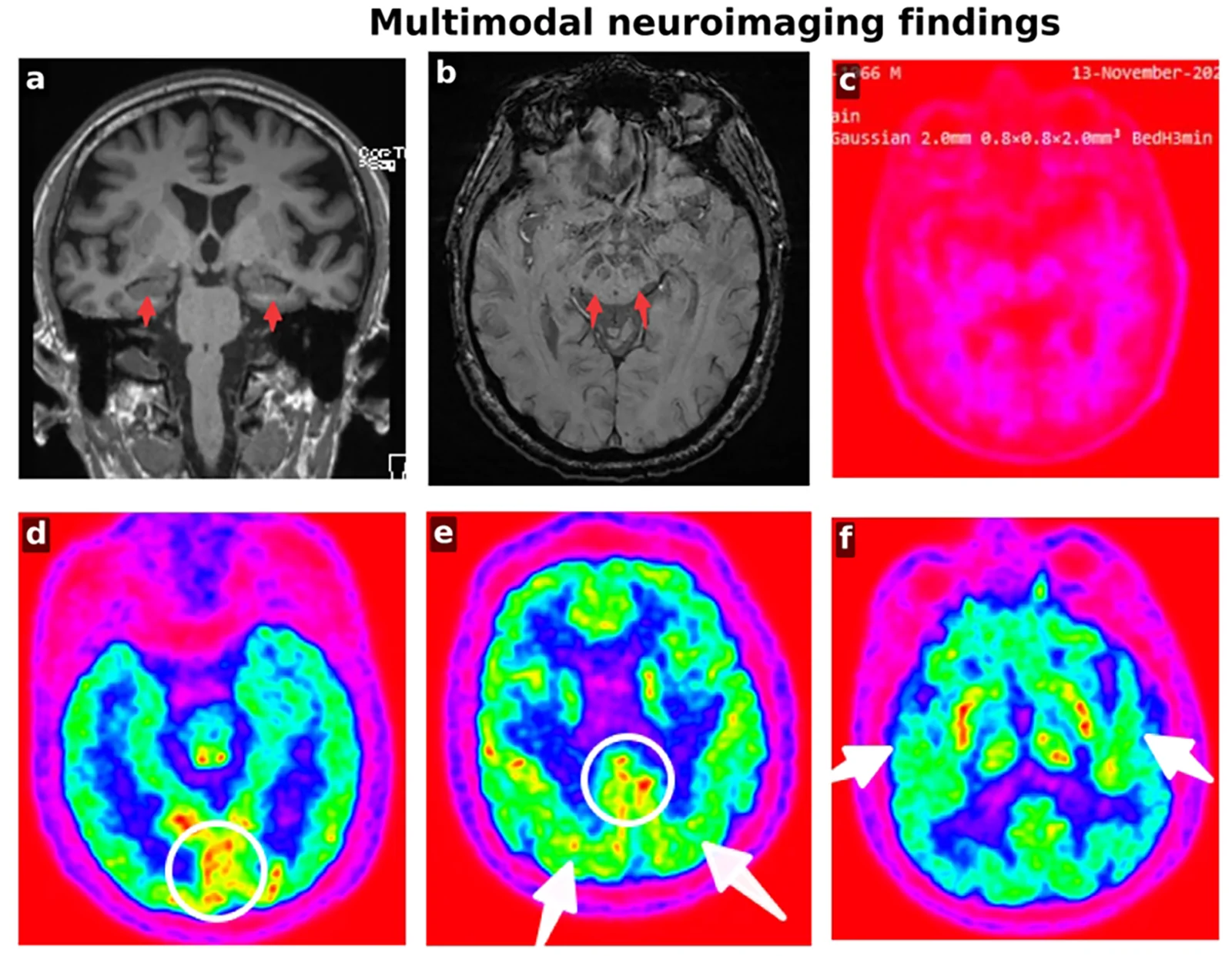
Multimodal neuroimaging findings. **(A)** Brain MRI showing medial temporal atrophy (score 1). **(B)** SWI demonstrating bilateral nigrosome-1 signal loss (“swallow tail sign” absent). **(C)** Amyloid-beta PET ([18F]florbetapir) showing no cortical tracer retention. **(D)** FDG-PET showing relative preservation of primary visual cortex (V1) (white circle). **(E)** Cingulate island sign (white circle: preserved posterior cingulate cortex metabolism; white arrows: bilateral occipital hypometabolism). **(F)** Lateral temporal hypometabolism (white arrows).

Key ancillary findings are summarized in **Table 2**. Notable structural and molecular findings included bilateral nigrosome-1 signal loss on susceptibility-weighted imaging (7, 8) (**Figure 2B**); occipital hypometabolism with preserved posterior cingulate metabolism (cingulate island sign)(**Figure 2E**) and relative primary visual cortex (V1) sparing(**Figure 2D**)on fluorodeoxyglucose positron emission tomography (FDG-PET) (9), with bilateral temporal hypometabolism also observed (**Figure 2F**); negative amyloid-beta PET (**Figure 2C**); and posterior diffuse slowing on electroencephalography. Cerebrospinal fluid (CSF) analysis revealed amyloid-beta42–907 pg/mL and amyloid-beta40 17,120 pg/mL (elevated), with an amyloid-beta42/amyloid-beta40 ratio of 0.05 (decreased; normal >0.1); total tau 84.03 pg/mL (decreased); and phosphorylated tau at threonine 181 24.96 pg/mL (decreased) (Enzyme-linked immunosorbent assay (ELISA) was conducted using an RT-6100 absorbance microplate reader. Rayto, Shenzhen, China). The discordance between decreased CSF amyloid-beta42 and negative amyloid-beta PET may reflect relatively low amyloid burden or spatial heterogeneity in DLB, resulting in insufficient PET visualization; this pattern does not support a typical Alzheimer’s disease biomarker profile (10).

**Table 2:** Chronological multimodal biomarker assessment and diagnostic category classification.

Time point	Examination	Key findings	Diagnostic category
June-July 2025	Comprehensive Geriatric Assessment	MMSE 23, MoCA 19, Barthel Index 100, IADL 4/8.	Supports basic criteria
June-July 2025	Brain MRI (3.0T): T1, SWI	Mild widening of perimesencephalic cisterns. Bilateral midbrain ‘swallow tail sign’ absent.	Exploratory marker
June-July 2025	EEG (16-channel)	Diffuse slowing in posterior parietal and occipital regions (2.5–4 Hz slow waves).	Supportive biomarker
June-July 2025	Cerebrospinal fluid	Aβ42–907 pg/mL/Aβ40 17,120 pg/mL ratio = 0.05 (decreased; normal: >0.1); total tau 84.03 pg/mL (decreased).	Exploratory marker
November 2025	FDG-PET	Diffuse hypometabolism in bilateral frontal, parietal, temporal, and partial occipital lobes; relative preservation of occipital V1 and posterior cingulate.	Supportive biomarker
November 2025	Aβ-PET	No cortical tracer retention (amyloid-negative).	Excludes Alzheimer’s disease
January 2026	Polysomnography	REM percentage 19%. REM sleep without atonia. Video-documented vocalizations and limb movements. **Supplementary Videos 1, 2**.	Indicative biomarker
March 2026	Brain MRI (3.0T): T1 (coronal)	Medial temporal atrophy score 1.	Supportive biomarker

Biomarker hierarchy is based on the 2017 DLB Consortium consensus criteria: indicative biomarkers possess high diagnostic specificity; supportive biomarkers assist differential diagnosis; exploratory markers require further multicenter validation and have not been incorporated into the 2017 guidelines. Elevated or decreased values are relative to each laboratory’s reference range.

SWI = susceptibility-weighted imaging; FDG-PET = [18F]-fluorodeoxyglucose positron emission tomography; Aβ-PET = [18F]-florbetapir amyloid-beta PET; RBD = rapid eye movement sleep behavior disorder; V1 = primary visual cortex.

Diagnostic Integration: The patient fulfilled four core clinical features, satisfying the 2017 DLB Consortium criteria for probable DLB (2). Ancillary investigations were arranged in a hierarchical differential diagnostic priority (**Table 2**): bilateral nigrosome-1 signal loss on susceptibility-weighted imaging indicated substantia nigra dopaminergic neurodegeneration; baseline RBD, corroborated by bed-partner history, fulfilled a core clinical feature and was subsequently objectified by polysomnography in January 2026; FDG-PET occipital hypometabolism with preserved posterior cingulate metabolism aligned with the characteristic DLB metabolic pattern; posterior EEG slowing supported posterior cortical dysfunction; and negative amyloid-beta PET combined with CSF biomarkers arguing against a typical Alzheimer’s disease profile substantially reduced the likelihood of Alzheimer’s disease as the dominant pathology.

Therapeutic Intervention: Given the patient’s poor response to the acute levodopa challenge test, together with a history of chronic hallucinations and hypersensitivity to antipsychotic medications, we did not proceed with a chronic sequential levodopa trial (11). The treatment regimen comprised (1) rasagiline 1 mg orally every morning for motor symptom amelioration (12) (2); donepezil 5 mg orally at bedtime targeting cholinergic deficits in DLB to improve cognition and neuropsychiatric symptoms (13, 14); and (3) agomelatine 25 mg orally at bedtime to regulate sleep-wake rhythm and ameliorate anxiety. This strategy avoided the risks of motor deterioration and excessive sedation associated with dopamine receptor blockade.

Follow-up and Outcomes: At 3 months, the MoCA score remained 19; UPDRS-III improved from 27 to 25; and the total Neuropsychiatric Inventory score decreased from 28 to 22 (hallucination subscore: 8-4). At 12-month follow-up, cognitive performance was maintained at baseline (MoCA 19), motor symptoms improved (UPDRS-III 27-25), auditory hallucination frequency declined from 2–3 episodes weekly to 1–2 monthly, and visual hallucinations became sporadic with retained insight into their unreal nature. Treatment adherence was excellent. His wife reported reduced stigma and anxiety after the patient understood the neurodegenerative etiology of his hallucinations. Non-pharmacological interventions included RBD-related sleep environment safety modifications and caregiver psychoeducation.

## 4 Discussion

The “auditory-predominant” chronology, with isolated auditory hallucinations preceding visual hallucinations by 4 years, is exceedingly rare in DLB (pure auditory hallucination incidence 0.6%) (3). It should be emphasized that this 0.6% refers to auditory hallucinations unaccompanied by visual hallucinations, whereas the auditory-priority sequence in this case (isolated auditory hallucinations antedating visual hallucinations by several years) represents an even rarer individual phenomenon that cannot be extrapolated as a DLB-specific prodromal marker. Okamoto et al. (15) previously reported a prodromal DLB case with Buddhist chanting hallucinations as the inaugural symptom, and Tsunoda et al. (16) observed that auditory hallucinations in DLB frequently co-occur with visual hallucinations, constituting a “soundtrack” phenomenon. The uniqueness of our case lies in the isolated emergence of auditory hallucinations years prior to visual hallucinations, suggesting that alpha-synuclein pathology may involve auditory-related networks early in the disease course; however, this remains a pathophysiological hypothesis requiring autopsy or longitudinal neuroimaging validation.

DLB hallucinations are closely linked to cholinergic-dopaminergic imbalance: cortical cholinergic denervation reduces sensory input weighting, whereas relatively preserved dopaminergic signaling amplifies internal perceptual priors, thereby generating hallucinations within a Bayesian perceptual inference framework (17). FDG-PET demonstrated bilateral temporal hypometabolism, possibly related to auditory processing network dysfunction; occipital hypometabolism with V1 sparing is consistent with the characteristic DLB metabolic pattern (9). However, establishing a direct causal link between metabolic abnormalities in specific brain regions and isolated auditory hallucinations remains speculative; their generation more likely involves cortical-subcortical cholinergic-dopaminergic network imbalance (17).

This misdiagnosis experience not only underscores the necessity of collaboration between geriatric psychiatry and neurology, but also highlights the value of isolated auditory hallucinations as a “warning signal” rather than a diagnostic criterion. The external hospital rendered a phenomenological diagnosis of schizophrenia and prescribed olanzapine, overlooking the following neurodegenerative red flags (1): non-commenting, non-commanding content of hallucinations-DLB hallucinations typically manifest as neutral or everyday conversations, distinct from the threatening, commanding content characteristic of schizophrenia (15, 16); and (2) prodromal RBD and autonomic dysfunction. Isolated auditory hallucinations per se lack diagnostic specificity; however, when accompanied by RBD, autonomic symptoms, or parkinsonian signs, neurodegenerative disorders should be prioritized to shorten diagnostic delay and avoid antipsychotic exposure.

The absence of dopamine transporter PET and 123I-metaiodobenzylguanidine scintigraphy did not compromise the probable DLB diagnosis. Follow-up MRI (March 2026) demonstrated a medial temporal atrophy score of 1 (**Figure 2A**), further arguing against an Alzheimer’s disease-dominant pathology. Multimodal investigations were primarily used to exclude Alzheimer’s disease and primary psychiatric disorders-the cingulate island sign on FDG-PET, posterior EEG slowing, and negative amyloid-beta PET collectively constituted an evidence chain differentiating DLB from Alzheimer’s disease (9, 10). This integrated approach of “clinical features as the cornerstone, objective examinations as ancillary” is particularly pertinent for resource-limited settings.

The acute severe adverse reaction induced by olanzapine represents a critical therapeutic warning in this case. Against the background of cholinergic deficiency in DLB, olanzapine’s antagonism of histamine H1 and alpha1-adrenergic receptors is markedly amplified: histamine H1 receptor blockade precipitates arousal maintenance failure (acute Epworth Sleepiness Scale score elevation), whereas alpha1-adrenergic receptor antagonism induces symptomatic orthostatic hypotension superimposed on pre-existing peripheral autonomic dysfunction. This “dual amplification effect” partially explains the poor olanzapine tolerance in DLB (4). Therefore, in middle-aged and elderly patients with new-onset neuropsychiatric symptoms, the presence of RBD, autonomic dysfunction, or parkinsonian signs should prompt DLB risk assessment before antipsychotic initiation. Cholinesterase inhibitors (e.g., donepezil, rivastigmine) should serve as the first-line therapeutic option for neuropsychiatric symptoms in DLB, with their efficacy in hallucination amelioration through enhanced cholinergic transmission having been validated in randomized controlled trials (13, 14).

Alternative strategies following olanzapine intolerance require careful efficacy-safety balancing. Quetiapine is frequently used for neuropsychiatric symptoms in DLB, yet randomized controlled trial evidence is mixed, and it may exacerbate sedation and orthostatic hypotension (18, 19). Clozapine is constrained by agranulocytosis risk (18). Pimavanserin, a selective serotonin 2A receptor inverse agonist lacking dopamine receptor binding, avoids motor symptom deterioration (19). Although approved by the U.S. Food and Drug Administration in 2016 for Parkinson’s disease psychosis, its efficacy and safety in DLB await confirmation in larger-scale studies. Given the patient’s prominent RBD and sleep rhythm disturbances, agomelatine 25 mg was empirically added to regulate sleep-wake rhythm; this agent does not block dopamine receptors, but evidence in DLB remains limited, necessitating hepatic function monitoring.

## 5 Patient perspective

The patient reported that early RBD symptoms were misinterpreted as nightmares, delaying medical consultation. The acute adverse reaction to olanzapine generated treatment aversion, postponing systematic evaluation. After understanding the neurodegenerative etiology of his hallucinations, his stigma markedly diminished and treatment adherence improved. His current predominant concern is the impact of long-term cognitive and motor decline on quality of life.

## 6 Limitations

This report represents a single-case observation; 12-month follow-up is insufficient to assess long-term progression and durable efficacy, and autopsy or histopathological confirmation is lacking. FDG-PET and amyloid-beta PET were performed 4 months after treatment initiation; the potential influence of medication on cerebral metabolism and tracer distribution cannot be entirely excluded. These investigations were subsequent supplementary examinations primarily for differential diagnosis, and the diagnostic basis rested on core clinical features at the initial visit rather than on these imaging results. The chronology of isolated auditory hallucinations preceding visual hallucinations is merely an individual phenomenon that cannot be inferred as a DLB-specific prodromal marker, their extended value requires validation in prospective cohort studies.

## Data Availability

The original contributions presented in the study are included in the article/**Supplementary Material**. Further inquiries can be directed to the corresponding author.
